# Short-form RON (sf-RON) enhances glucose metabolism to promote cell proliferation via activating β-catenin/SIX1 signaling pathway in gastric cancer

**DOI:** 10.1007/s10565-020-09525-5

**Published:** 2020-05-12

**Authors:** Ziliang Wang, Yufei Yang, Shuang Hu, Jian He, Zheng Wu, Zihao Qi, Mingzhu Huang, Rujiao Liu, Ying Lin, Cong Tan, Midie Xu, Zhe Zhang

**Affiliations:** 1 Department of Medical Oncology and Cancer Institute, Fudan University Shanghai Cancer Center, Shanghai, 200032 China; 2 Department of Obstetrics and Gynecology, Xinhua Hospital Affiliated to Shanghai Jiaotong University School Medicine, Shanghai, 200092 China; 3 Department of Gynecological Oncology, Fudan University Shanghai Cancer Center, Shanghai, 200032 China; 4 Department of Pharmacy, Eye & Ent Hospital of Fudan University, Shanghai, 200031 China; 5 Department of Oncology, Shanghai Medical College, Fudan University, 270 Dong’an Road, Shanghai, 200032 China; 6 Huadong Hospital Affiliated to Fudan University, Shanghai, 200040 China; 7 Department of Pathology, Fudan University Shanghai Cancer Center, Shanghai, 200032 China; 8 Department of Pathology, Fudan University Shanghai Cancer Center, 270 Dong’an Road, Shanghai, 200032 China

**Keywords:** sf-RON, β-Catenin, SIX1, Glucose metabolism, Gastric cancer

## Abstract

Recepteur d’origine nantais (RON) has been implicated in cell proliferation, metastasis, and chemoresistance of various human malignancies. The short-form RON (sf-RON) encoded by RON transcripts was overexpressed in gastric cancer tissues, but its regulatory functions remain illustrated. Here, we found that sf-RON promoted gastric cancer cell proliferation by enhancing glucose metabolism. Furthermore, sf-RON was proved to induce the β-catenin expression level through the AKT1/GSK3β signaling pathway. Meanwhile, the binding sites of β-catenin were identified in the promoter region of SIX1 and it was also demonstrated that β-catenin positively regulated SIX1 expression. SIX1 enhanced the promoter activity of key proteins in glucose metabolism, such as GLUT1 and LDHA. Results indicated that sf-RON regulated the cell proliferation and glucose metabolism of gastric cancer by participating in a sf-RON/β-catenin/SIX1 signaling axis and had significant implications for choosing the therapeutic target of gastric cancer.

**Electronic supplementary material:**

The online version of this article (10.1007/s10565-020-09525-5) contains supplementary material, which is available to authorized users.

## Introduction

Gastric carcinoma (GC) is one of the most common gastrointestinal cancers and ranks a third cause of cancer-related death worldwide (Siegel et al. 2020). In the past years, although considerable attention was paid to early screening and surgical techniques, patient mortality, due to recurrence, metastasis, and chemotherapy resistance, remains high (Van Cutsem et al. 2016; Necula et al. 2019). Therefore, the development of early diagnosis maker, as well as therapeutic target, remains an ongoing challenge and it is worthwhile devoting much effort to this area.

Aerobic glycolysis (Warburg effect) is a biochemical fingerprint of cancer cells that represents one of the “hallmarks of cancer.” Although it is a much more inefficient way for ATP production compared to oxidative phosphorylation, this rapidly satisfies the energy requirement of cancer cells for quick proliferation (Sun et al. 2018). Cumulative evidence supported gastric cancer as a metabolic disease and pointed out that ectopic expression of certain metabolic enzymes in GC cells could enhance apoptosis and suppress cell proliferation and glucose metabolism (Li et al. 2013), which caused the worse prognosis in GC (Sun et al. 2019). Hence, GC has close relations with glucose metabolism, and the glucose metabolism level in cancer cells may be utilized to determine the clinical stage of patients with GC (Kaneko et al. 2015).

Recepteur d’origine nantais (RON) belongs to the c-Met family of scatter factor receptors, and is encoded by the MST1R gene in human entities (Song et al. 2012). Mature human RON protein (180 kDa) is a sort of heterodimer composed by an α-chain (40 kDa) and a transmembrane β-chain (150 kDa) (Ling et al. 2017). The extracellular domains of RON are involved in phosphorylation, receptor dimerization, and ligand binding (Andrade et al. 2017), and the intracellular domains of RON are performing as recruitment sites for transducers and adaptors (Suthe et al. 2018). RON is highly expressed in multiple epithelial carcinomas involving the lung, liver, gastrointestinal tract, skin, etc. (Chang et al. 2015) and can halt cell cycle and promote the cancer cell proliferation (Han et al. 2017; Chen et al. 2017, 2018; Zhou et al. 2019). RON transcripts were encoded into a full-length protein (f-RON) and a short-form protein (sf-RON) (Faham and Welm 2016). Sf-RON is upregulated and implicated in various human tumor entities (Xu et al. 2017b; Moxley et al. 2016; Liu et al. 2011). Chang, K. and colleagues showed that activation of RON signaling pathways reduced the inhibitory effect of MET inhibitors on proliferation and migration pancreatic cancer cells (Chang et al. 2015). Interestingly, of the two kinds of RON, only the overexpression of sf-RON, rather than f-RON, conferred resistance to MET inhibitor (Zhuang et al. 2015). Furthermore, sf-RON was demonstrated overexpressed in the MET-positive GC tissue samples (Wu et al. 2015). However, the mechanism how sf-RON regulated GC cell proliferation still remains unknown.

In GC, overexpression of RON/RONDelta160 can promote the cell growth and migration in GC through inducing the β-catenin expression, which establishes a link between β-catenin and RON, and adding new evidence on the mechanism by which they contribute to GC progression (Zhou et al. 2019). Furthermore, scientists elucidated that loss of N-bisected glycosylation on RON caused by variable splicing directly leads to tumorigenesis and poorer prognosis in GC (Koh et al. 2019). Meanwhile, RON was identified to work as a promoter in the regulation of glucose metabolism in the liver (Li et al. 2015). Although which form should participate in remains unknown, RON has a closed correlation to glucose metabolism.

Our study showed the role of sf-RON in the glucose metabolism in GC and validated that sf-RON enhanced the cell proliferation and glucose metabolism of gastric cancer cells more than RON did. Mechanistically, we demonstrated that sf-RON activated the β-catenin/SIX1 signaling pathway, regulating the expression of key glycolytic genes involved in GC. Therefore, our results certified the regulatory functions of sf-RON/β-catenin/SIX1 signaling axis on the cell proliferation and glucose metabolism of GC.

## Materials and methods

### Tissue samples and construction of tissue microarray

The formalin-fixed paraffin-embedded (FFPE) tissues of 137 patients with GC who had undergone surgery at Fudan University Shanghai Cancer Center (FUSCC) between June 2012 and January 2017 were included for immunohistochemistry investigations. Supplementary Table 1 shows the clinicopathological features of the patients. None of the patients received preoperative drug therapy. The follow-up interval was from the date of diagnosis to the date of the latest clinical investigation, disease progression, or death. The present study was ethically approved by the Ethics Committee of FUSCC, and all patients provided written informed consent.

Following histologic examination by an experienced pathologist, the locations of all foci were determined. Cores (1 mm in diameter) of the FFPE blocks were obtained for tissue microarray (TMA) construction. Each case has two separate cores to exclude the heterogeneity of tumors. Two suitable cancer foci and 2 adjacent foci of normal epithelial, randomly selected from137 cases, were inserted into the recipient paraffin blocks.

### Immunohistochemistry assay

The antibody information for immunohistochemistry (IHC) was all purchased from Abcam company and summarized as follows: β-catenin (ab32572, 1:100 dilution), SIX1 (ab211359, 1:100 dilution), GLUT1 (ab115730, 1:100 dilution), and LDHA (ab226016, 1:100 dilution). The immunoreactive score (IRS) is the product of the intensity of the immunoresponse and the percentage of cancer cell staining, as described previously (Gan et al. 2017). The assessment of the protein expression was defined based on the IRS as low (≤ 1+) and high (> 2 + to ≤ 3+).

### Cell culture, plasmid construction, and viral infection

Human GC cell lines GTL-16 and MKN-45 and the recombinant plasmid pWPXL-sf-RON were preserved and used in our laboratory as previously described (Wu et al. 2015). The promoter sequence of SIX1 (− 3000 ~ +84 bp) was sub-cloned into pGL3-basic vector and then co-transfected with phRl-TK vector into cells for establishment of the recombinant plasmid, as previously described (Cao et al. 2019). The potential binding sites of β-catenin in the promoter of SIX were analyzed using UCSC online database (http://genome.ucsc.edu/).

The target sequences of siRNAs (Transheep, Shanghai, China) for target gene were sf-RON-si1: 5′-CTGACTGTGTGGGTATCAA-3′ and sf-RON-si2: 5′-GACTGTGTGGGTATCAACG-3′; for RON-si1: 5′-CCAGTACAGGTCACTGCAT-3′ and RON-si2: 5′-GCACAATGGATGGGCGTAT-3′.

### RNA-seq data analysis

We enrolled the gene expression profile in sf-RON positive GC tissue samples (*n* = 26) and RON-positive GC tissue samples (*n* = 13), as well as sf-RON overexpression (OE) and control cell samples for RNA-seq data analysis. Total RNA (1 μg) was isolated from GTL-16 cells and submitted for the construction of the RNA libraries. RNA sequencing was performed and analyzed as previously described (Cao et al. 2019). Gene set enrichment analysis (GSEA) using H hallmarks sets was used for gene functional annotation (Kim et al. 2008).

### ^18^F-FDG positron emission tomography/computed tomography scan

Whole-body FDG positron emission tomography/computed tomography (PET/CT), OCR, and EACR assays were performed as previously described (Cao et al. 2019).

### Western blotting assay, RNA isolation, and quantitation

Western blotting and reverse transcription quantitative real-time polymerase chain reaction (RT-qPCR) were used for molecules detection and performed as previously described (Wu et al. 2015). Antibodies against HK2 (22029-1-AP,), AKT1 (10176-2-AP), LDHA (19987-1-AP, ), GLUT1 (21829-1-AP), and GSK3β (22104-1-AP) all purchased from Proteintech; β-Actin (#4970), phosphor-AKT1 (Ser473, #4060), and phosphor-GSK3β (Ser9, #5558), all purchased from Cell Signaling Technology, were used to assess the expression of an individual protein. Secondary antibodies were used at 1:5000 dilutions. The primer sequences for RT-qPCR involved in this study were shown in Supplemental Table 2.

### Immunofluorescence, cell viability, glycolysis, and colony formation assay

Immunofluorescence, cell viability, glycolysis, and colony formation assay were performed as previously described (Gan et al. 2017).

### Chromatin immunoprecipitation and luciferase reporter assay

Chromatin immunoprecipitation (ChIP) and luciferase reporter assays were used for the detection of promoter regulation and performed as previously described (Gan et al. 2018).

### Xenograft experiments

Animal experiments were approved by the Shanghai Medical Experimental Animal Care Commission. Briefly, male BALB/c nude mice (4–6 weeks) were subcutaneously injected with cells (10^7^ cells in 0.1 mL PBS). Subcutaneously xenograft model was performed as previously described (Xu et al. 2017a). PET/CT scan was performed for the evaluation of glucose uptake and calculating the SUVmax value when the average tumor volume of all the mice reached 100 mm^3^. Sections of the tumors were cut and subjected to immunohistochemical staining as previously described (Gan et al. 2018).

### Statistical analysis

Statistical analyses were performed with SPSS 24.0 (SPSS Inc., Chicago, IL). All experiments were repeated in triplicate, and the data are presented as the mean ± SDs. Comparisons between groups were performed with a paired *t* test or one-way ANOVA, as appropriate. The clinical data were analyzed using *χ*^2^ test and the Spearman correlation coefficient. The Kaplan–Meier method with log-rank analysis was used to estimate the DFS and OS. The multivariate analysis with the Cox proportional hazards model enrolled all variables with a *P* value less than 0.05 in univariate analysis. Two-tailed *P* value less than 0.05 was considered to indicate a statistical significance.

## Results

### sf-RON expression is closely associated with glucose metabolism in GC patients

**Fig. 1:**
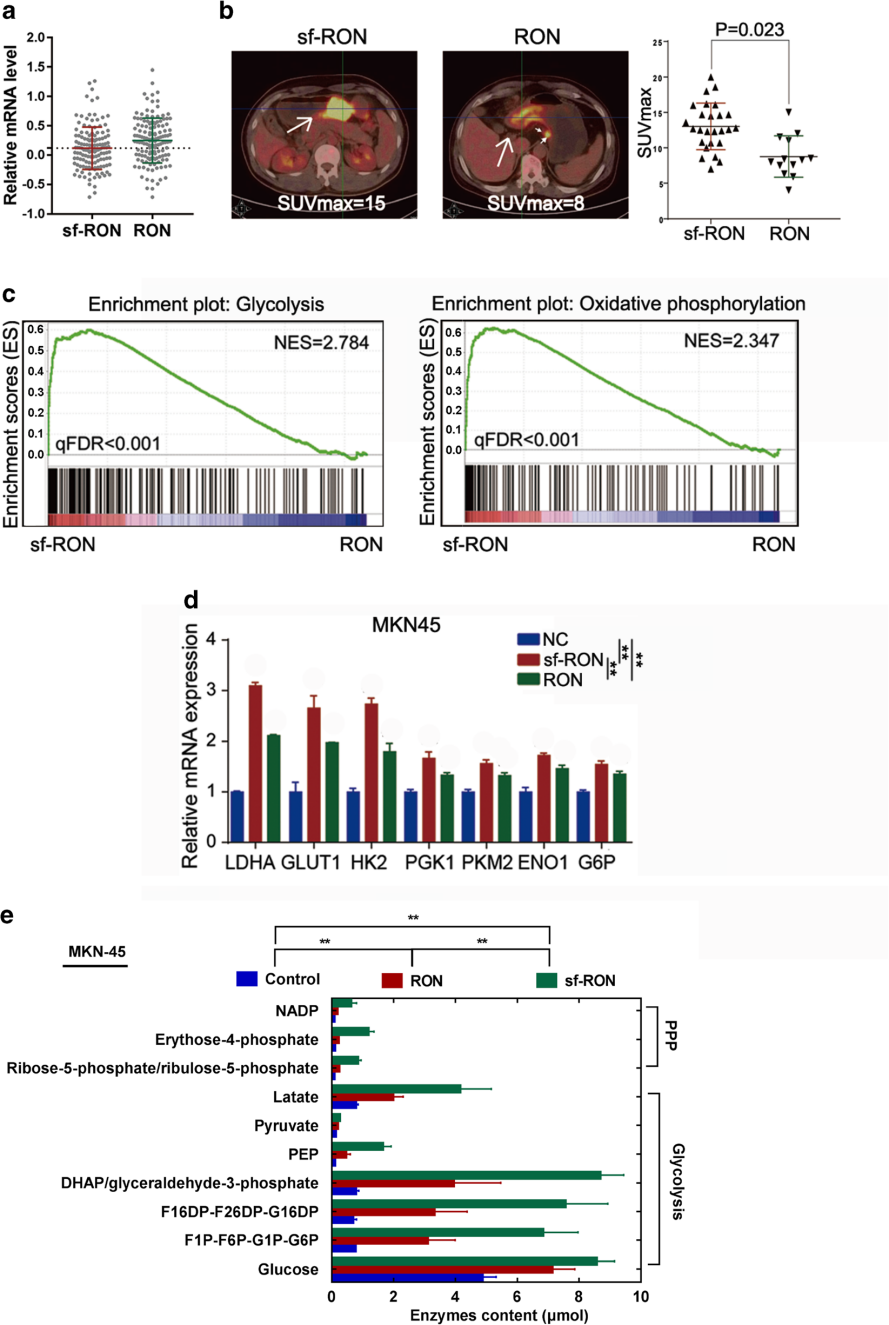
sf-RON was closely related to the glucose metabolism in GC patients. **a** The RT-qPCR result of sf-RON or RON mRNA level in all 173 patients with GC. The dotted line represents the mean value of sf-RON level was selected as the cutoff value. **b** The representative ^18^F-FDG-PET-CT images from GC patients with sf-RON (left) or RON (right) expression (all × 200) and SUVmax of gastric cancer patients with sf-RON and RON expression (*n* = 39). **c** GSEA analysis was performed using the tissues with high sf-RON and the corresponding controls. The signature was defined by genes with significant expression changes in glycolysis, and oxidative phosphorylation. Gene expression profiling was performed with RNA sequencing. **d** qRT–PCR analysis of indicated genes in the gastric cancer tissues with high sf-RON OE and the corresponding controls. Data are shown as mean ± SD. Significance was calculated using Student’s *t* test. * *P* < 0.01. **e** Relative enzymes content of intermediates in gastric cancer tissues compared with those in para-carcinoma tissues (*n* = 5 samples of each group). Blue and red indicate downregulation or upregulation, respectively

We firstly detected both sf-RON and RON expression in all 137 GC samples by RT-qPCR and selected the mean value (0.108) of sf-RON level as the cutoff value, then 64 cases showed high sf-RON expression, and 73 patients showed high RON expression; thus, these cases were divided into sf-RON group (*n* = 64) and RON group (*n* = 73, Fig. 1a). Among these patients, 39 had been undergone preoperative PET/CT scan, and we found that 26 patients only belonged to the sf-RON group, and 13 patients only belonged to the RON group. We then compared the metabolic activity of each lesion by analyzing the maximum standardized uptake value (SUVmax) and found that patients of RON group showed significantly lower SUVmax values than those in sf-RON group (*P* = 0.023, Fig. 1b), which indicates that compared with RON, sf-RON might have more close correlation with glucose metabolism in gastric patients. We further performed RNA sequencing using the 39 tissues from the sf-RON group (*n* = 26) and RON group (*n* = 13). GSEA results showed that glycolysis and oxidative phosphorylation (OXPHOS) pathways were enriched in the gastric tissues in the sf-RON group (Fig. 1c), which was validated by qRT-PCR assay and showed that overexpression of sf-RON and RON significantly upregulated glycolysis and OXPHOS related key enzymes in MKN-45 GC cell lines; interestingly, sf-RON exhibit strong promoting role than RON (Fig. 1d). To further identify the alterations of glucose metabolism in GC cells, we performed an analysis and found that pentose phosphate pathway (PPP) and glycolysis intermediates were obviously upregulated in GC tissues expressing high sf-RON, compared with the control group and RON group (Fig. 1e, Supplementary Table 3). These results illustrated that sf-RON was closely related with the progression and glucose metabolism in GC patients.

### sf-RON is a key promoter of glycolysis-mediated cell growth in GC

**Fig. 2:**
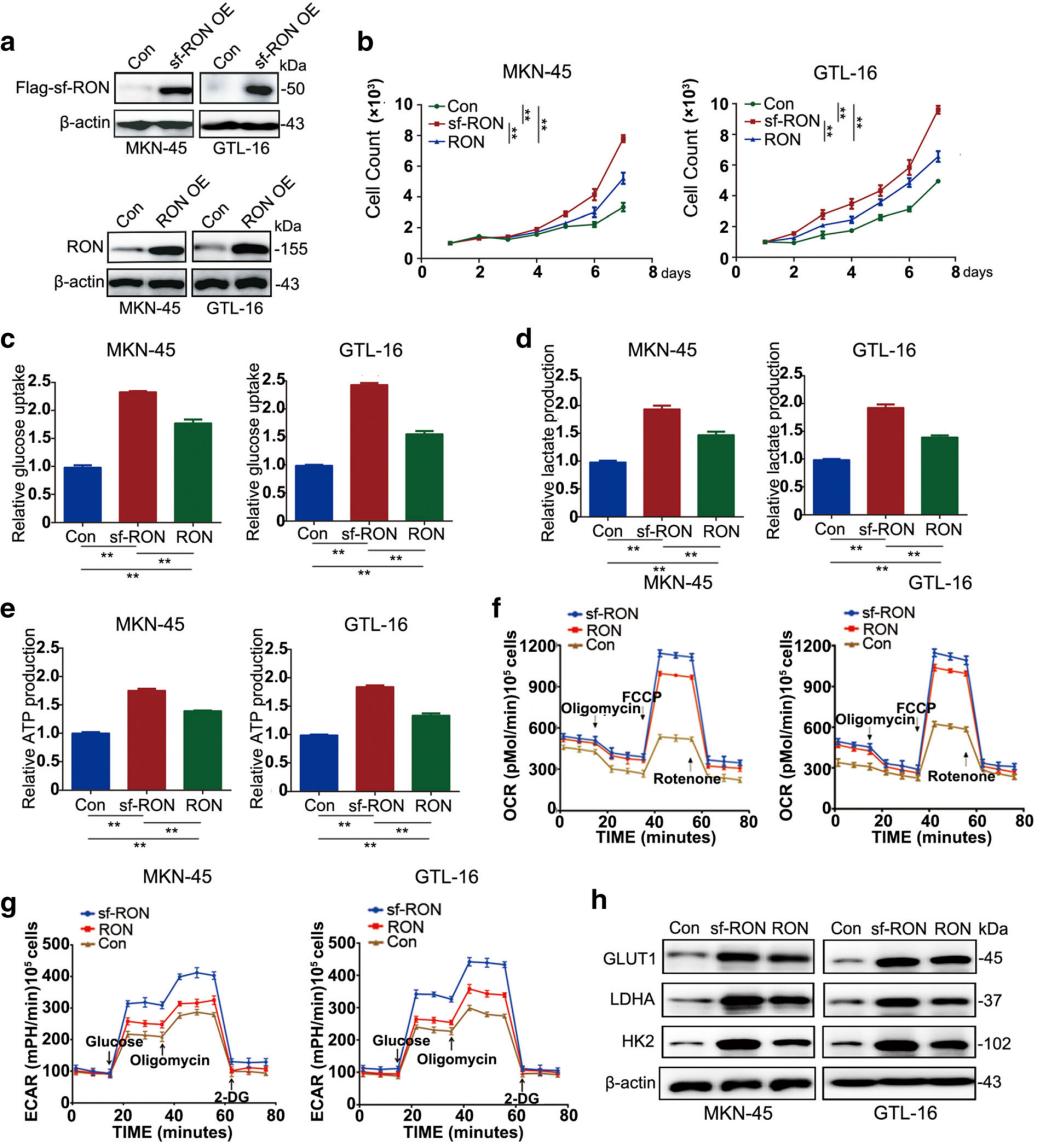
sf-RON promotes cell proliferation via enhancing glycolysis in GC cells. **a** Overexpression effects of sf-RON and RON in MKN-45 and GTL-16 cells were detected by western blotting assay. **b** Overexpression of sf-RON promoted cell growth in MKN-45 and GTL-16 cell lines compared with control cells using CCK-8 kit. **c–e** sf-RON enhanced the glucose consumption, glucose uptake, lactate uptake, and ATP uptake dramatically in MKN-45 and GTL-16 cell lines when compared with controls. **f** sf-RON increased the extracellular acidification rate (ECAR) and oxygen consumption rate (OCR) dramatically in gastric cells when compared with controls. Error bars = 95% CIs. The experiments were repeated three times, and a representative experiment is shown. **g** sf-RON induced the expression levels of GLUT1, LDHA, and HK2 by western blotting assay and qRT-PCR in MKN-45 and GTL-16 cells. **P < 0.01. **h** Expression of indicated protein in MKN-45 and GTL-16 cells were detected by western blotting assay

To determine the regulatory role of sf-RON in GC cells, we selected two GC cell lines GTL-16 and MKN-45 for the overexpression of sf-RON and RON and validated the protein expression levels by Western blot (Fig. 2a). We found that the overexpression of sf-RON and RON promoted the proliferation of GC and the number and size of the colonies; interestingly, sf-RON showed the stronger promoting effect in contrast to RON (Fig. 2a, Supplementary Figure 1). We then detected whether sf-RON-induced cell growth was mediated by glucose metabolism activation. The glycolysis results demonstrated that the glucose uptake and consumption, lactate and ATP production, ECAR, and OCR were all significantly increased after introduction of sf-RON compared with controls, consistently with the cell proliferation test; sf-RON also showed stronger facilitating effect on GC cell glucose metabolism than RON (Fig. 2c–g). The above results were further verified by assessing the molecular features in glucose metabolism; we found that the overexpression of sf-RON obviously upregulated the GLUT1, LDHA, and HK2 expression level of MKN-45 and GTL-16 cell lines, comparing the RON group and the normal counterparts (Fig. 2h). We also silenced specifically RON, sf-RON, and both together in MKN-45 and GTL-16 cell lines, which showed that knockdown of specifically sf-RON and both together decreased the glucose metabolism and cell proliferation more than knockdown of specifically RON, and compared with the specifically sf-RON silenced group, knockdown of both sf-RON and RON together significantly downregulated the glucose metabolism and cell proliferation (Supplementary Figure 2). The above results together indicated the promoting the role of sf-RON on glycolysis-mediated cell proliferation in GC cells.

### sf-RON promotes glycolysis-mediated cell progression through activating β-catenin signaling pathway

**Fig. 3:**
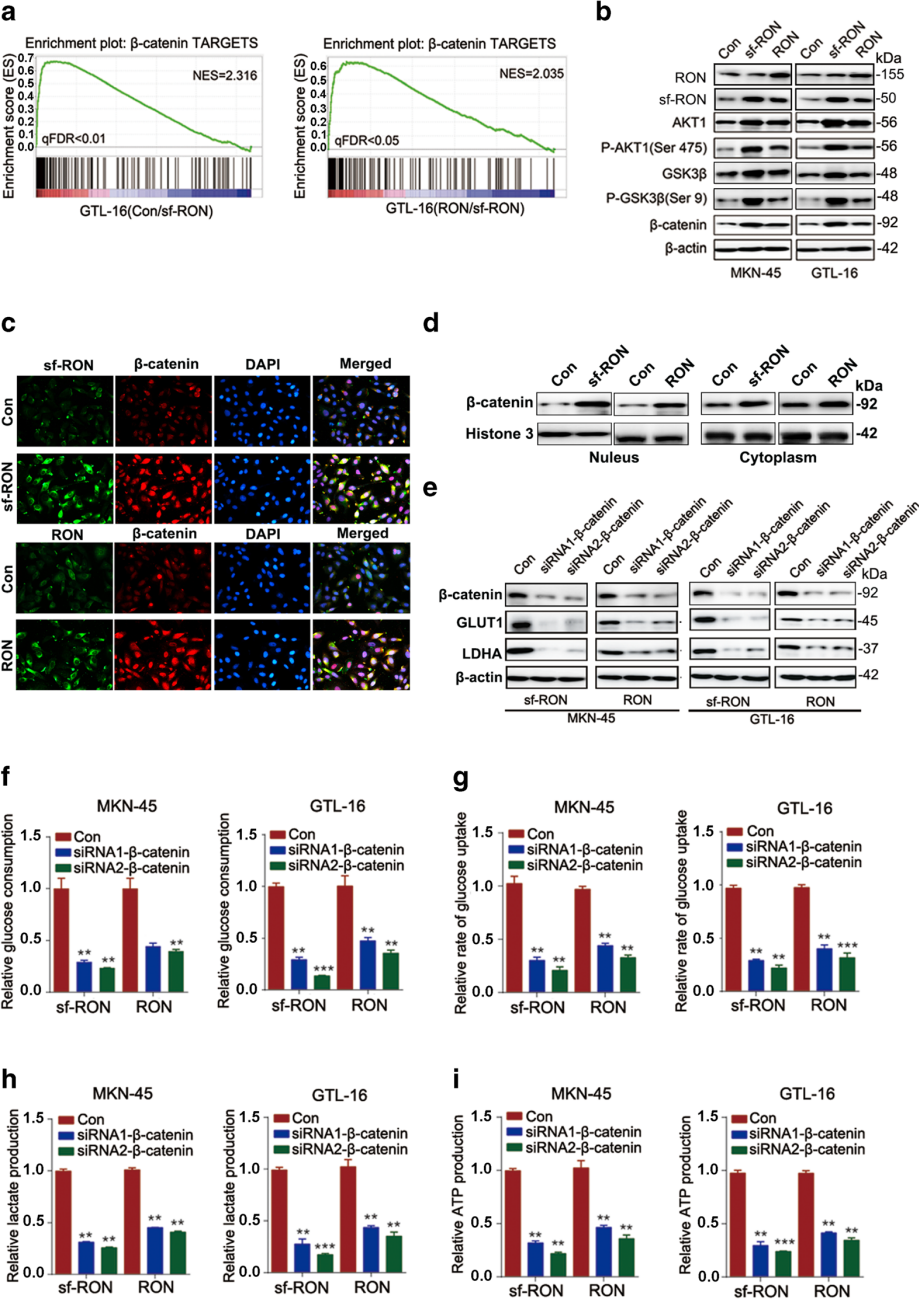
sf-RON regulated cell glycolysis by targeting β-catenin signaling pathway in GC cells. **a** GSEA analysis showed high enrichment of β-catenin pathway from RNA-sequencing data. **b** Overexpression of sf-RON obviously induced the expression levels of the members of β-catenin pathway, including AKT, P-AKT(Ser 473), GSK3β, and P-GSK3β(Ser 9). **c** Immunofluorescence assay showed that overexpression of sf-RON and RON promoted the expression of β-catenin in cell nucleus of MKN-45 cells. **d** The western blot result that the subcellular localization of β-catenin in the existence of RON or sf-RON. **e** Silencing of β-catenin decreased the expression of GLUT1 and LDHA in sf-RON and RON-overexpressing gastric cells detected by western blotting assay. **f–i** Silencing of β-catenin decreased the glucose consumption, glucose uptake, lactate uptake, ATP, and NADPH uptake dramatically in MKN-45 and GTL-16 cells with the overexpression of sf-RON. ** *P* < 0.01

sf-RON is known to activate several signaling pathways. To further illuminate the molecular mechanism of sf-RON in promoting glycolysis-mediated GC cell growth, we analyzed the potential enrichment pathways from RNA-sequencing data. Compared with the control and RON-overexpression cells, we observed the strong activation of β-catenin targets in sf-RON-overexpressing cells (Fig. 3a). According to western blot results, we found that overexpression of sf-RON increased expression levels of β-catenin, while RON showed a slighter promoting impact on β-catenin (Fig. 3b). To clarify the influence of RON and sf-RON on the subcellular localization of β-catenin, we performed immunofluorescence and captured images of β-catenin via confocal microscopy, which showed that overexpression of sf-RON and RON decreased the β-catenin level in the nucleus (Fig. 3c). The western blot analysis result also showed consistent results (Fig. 3d).

To identify whether β-catenin was a critical downstream molecule of sf-RON, we performed further experiments to disclose the correlation between sf-RON and β-catenin. We found that the impact of sf-RON on cell proliferation and glycolysis was eliminated by silencing of β-catenin in GTL-16 and MKN-45cells. More specifically, the silencing of β-catenin significantly rescued the promotive effect of sf-RON on the colony formation (Supplementary Figure 1) and the expression of glycolysis-related proteins (Fig. 3e). In terms of glucose metabolism, the silencing of β-catenin in the existence of sf-RON and RON-overexpressing GC cells partially reversed the change of glucose consumption (Fig. 3f), glucose uptake (Fig. 3g), lactate production (Fig. 3h), and ATP uptake (Fig. 3i). Above all, these findings demonstrated that sf-RON might boost GC progression by activation β-catenin expression.

### sf-RON/β-catenin signaling promote glycolysis partly by targeting SIX1 in GC

**Fig. 4:**
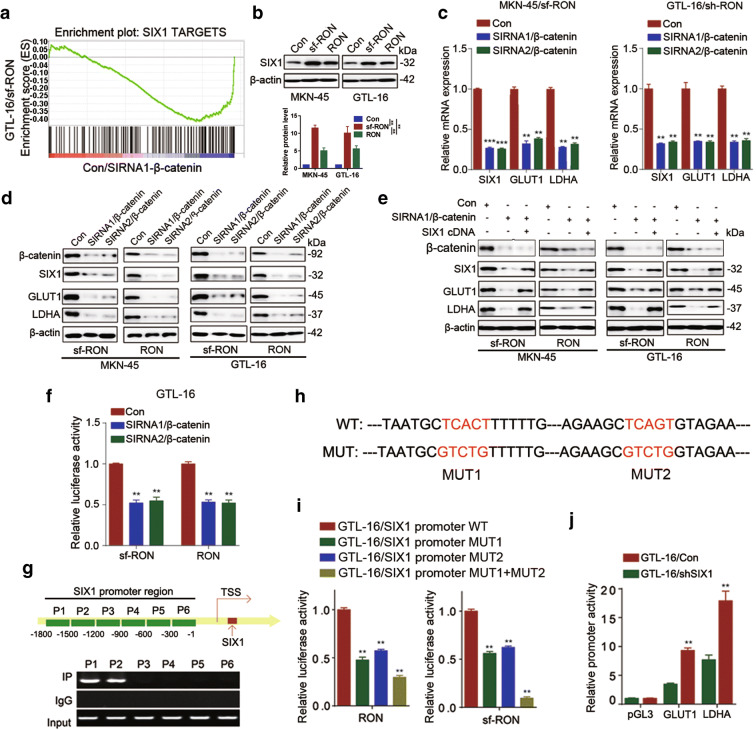
SIX1 was a direct target of sf-RON/β-catenin to regulate glycolysis in gastric cancer cells. **a** GSEA showed high enrichment of the SIX1 pathway from RNA sequencing data. **b** Western blotting showed that the expression level of SIX1 was enhanced in sf-RON-overexpressing gastric cancer cells, compared with RON-overexpressing and negative control cells. **c**, **d** Silencing of β-catenin reduced the expression level of SIX1 in sf-RON-overexpressing and RON-overexpressing gastric cancer cells detected by western blotting and qRT-PCR assay. **e** SIX1 rescued the silencing of β-catenin on the expression of GLUT1 and LDHA in gastric cells detected by western blotting assay. **f** Luciferase reporter assay was used to detect the regulation of β-catenin on the promoter activity of SIX1. ** *P* < 0.01. **g**, **h** PCR results of ChIP analysis showed that β-catenin bound to the SIX1 gene promoter region and the map of β-catenin binding sites in the promoter region of SIX1. **i** Luciferase reporter assay was used for the detection of β-catenin mutant sites in the promoter region of SIX1. ** *P* < 0.01. **j** Luciferase reporter assay showed that silencing of SIX reduced the promoter activity of GLUT1 and LDHA. ** *P* < 0.01

To further determine the downstream target of sf-RON/β-catenin signaling pathway, we analyzed the potential enrichment pathways from RNA-sequencing data, which showed high enrichment of SIX1 targets in the RON-overexpression and β-catenin-silenced cell compared with the control (Fig. 4a). SIX1 may be the potential target of sf-RON/β-catenin signaling pathway. To probe into its role in sf-RON-mediated glycolysis, we detected the SIX1 expression level in GC cells with overexpression of Ron and sf-RON (Fig. 4b). As expected, SIX1 was upregulated when overexpressing sf-RON or RON. In addition, the silencing of β-catenin downregulated the SIX1 levels, as well as the expression level of glycolytic protein (GLUT1 and LDHA) (Fig. 4c, d). While the introduction of SIX1 cDNA rescued the downregulation of the LDHA and GLUT1 expression levels induced by the silencing of β-catenin (Fig. 4e). Given that β-catenin is a well-known transcriptional factor, we next detected whether β-catenin transcriptional regulated the expression of SIX1. The detection of dual-luciferase reporter gene assay revealed that the silencing of β-catenin caused the decrease of SIX1 promoter activity (Fig. 4f). To determine the exact β-catenin binding sites on the promoter region of SIX1, we performed chromatin immunoprecipitation (ChIP) and confirmed that there were two β-catenin binding regions (Fig. 4g). Further luciferase assay showed that mutation of both binding sites decreased luciferase activity (Fig. 4h, i). As a key transcription factor, SIX1 has been implicated in the glucose metabolism that promotes glycolysis and tumor growth by stimulating glycolytic genes (such as GLUT1, ENO1, and LDHA) transcription (Li et al. 2018). To further elucidate the function of SIX1, we induced siRNA of β-catenin and SIX1 cDNA into sf-RON-overexpressing cells, and found that knockdown of β-catenin partly attenuated the promoting role of RON and sf-RON on GLUT1 and LDHA expression levels, while SIX1-overexpressing can partly rescue this phenomenon (Supplementary Figure 3A-B). Meanwhile, SIX1-silencing caused the decreasing of the promoter activity of both GLUT1 and LDHA (Fig. 4j). Functionally, β-catenin-silencing partly attenuated the promoting role of RON and sf-RON on the glucose metabolism, which was partly rescued by SIX1-overexpressing, compared with the control groups (Supplementary Figure 3C-F**)**.

### sf-RON promotes the progression of GC in vivo

**Fig. 5:**
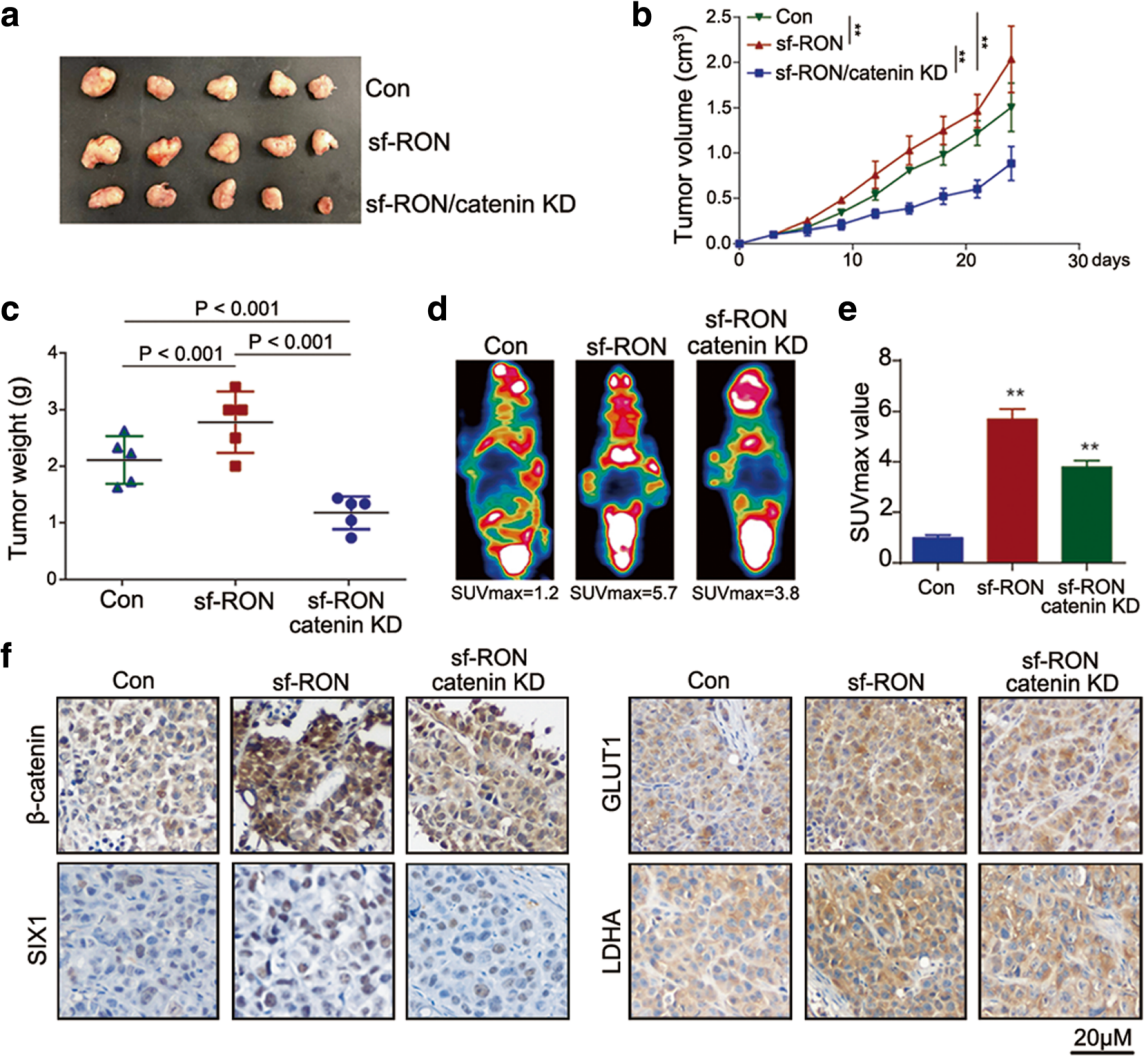
sf-RON promoted tumor growth by regulating β-catenin/SIX1 signaling in vivo. **a** Representative image of nude mice bearing tumors formed by sf-RON, sf-RON/β-catenin KD (Knockdown) and their control cells. **b** The average tumor volume of nude mice bearing tumors formed by sf-RON, sf-RON/β-catenin KD and their control cells. ** *P* < 0.01. **c** The average tumor weight of nude mice bearing tumors formed by sf-RON, sf-RON/β-catenin KD, and their control cells. **d** Representative image of PET-CT was used for the detection of glucose uptake in sf-RON, sf-RON/β-catenin KD xenografts, and their controls. **e** Average SUVmax values of nude mice bearing tumors. ** *P* < 0.01. **f** Immunohistochemical staining of β-catenin, SIX1, GLUT1, and LDHA in the sf-RON, sf-RON/β-catenin KD, and control tissues (magnification × 200)

Next, we tested the effects of sf-RON in vivo xenograft mice model. According to Fig. 5a–c, the overexpression of sf-RON accelerated the tumor growth speed and increased both the overall volume and weight of the tumor in vivo, and the knockdown of β-catenin attenuated the promoting effect of sf-RON on the xenograft tumor growth. In addition, PET-CT analysis showed an obvious higher SUVmax value as a result of increasing glucose uptake in sf-RON-overexpression group (Fig. 5d, e), which was attenuated by the knockdown of β-catenin. Immunohistochemistry assay showed that sf-RON enhanced the expression of β-catenin, as well as SIX1, GLUT1, and LDHA in xenograft tissues, which were rescued by the knockdown of β-catenin (Fig. 5f). To prove that SIX1 is the effector of the sf-RON/β-catenin pathway, we had silenced SIX1 in GC overexpressing RON and sf-RON and found that silenced SIX1 can partly attenuate the promoting role of RON and sf-RON in the glucose metabolism and cell proliferation (Supplementary Figure 3).

### Expression of sf-RON, β-catenin, and SIX1 is associated with survival of patients with GC

**Fig. 6:**
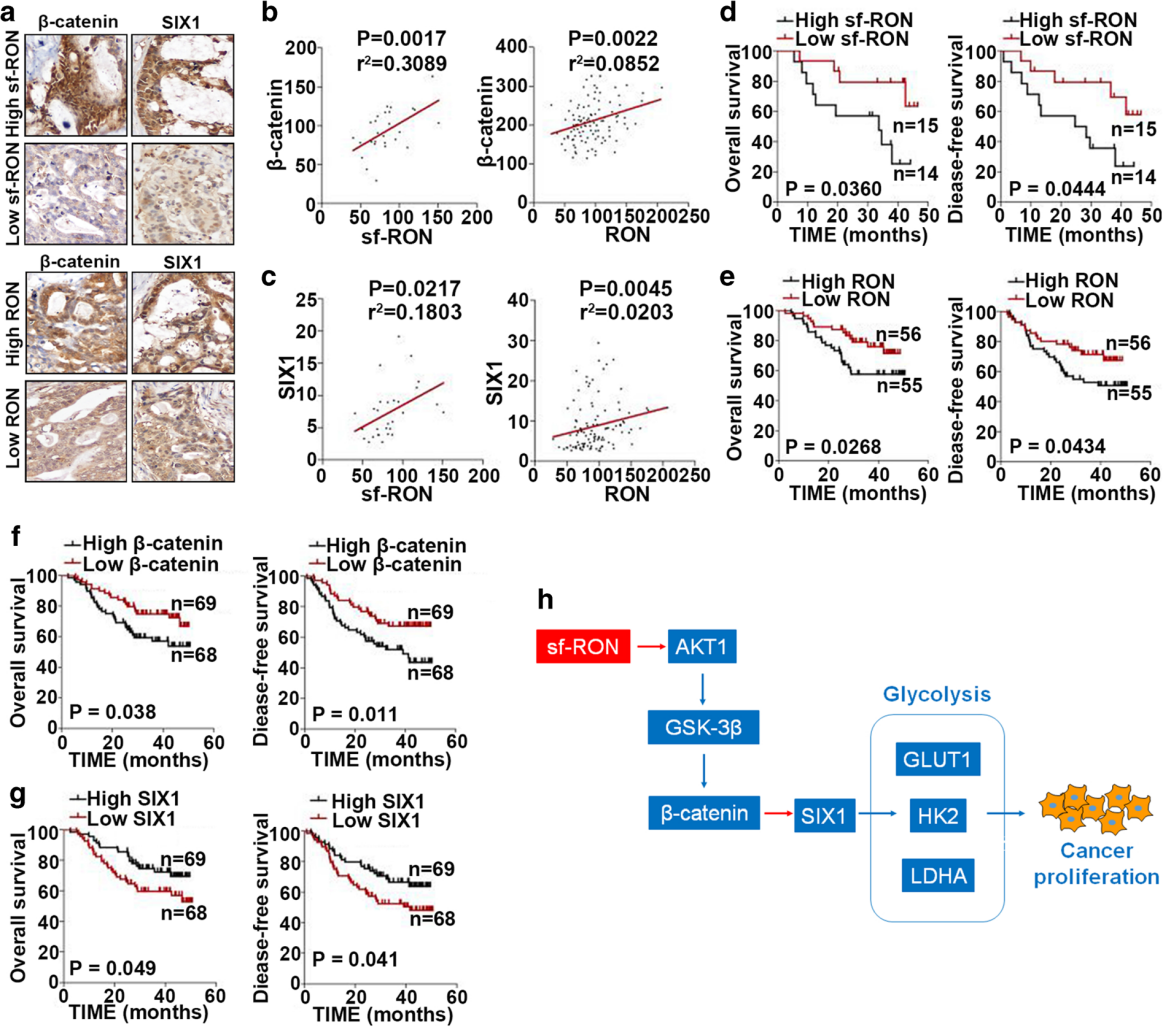
The associations among the expression of sf-RON, RON, β-catenin and SIX1, and survival of gastric cancer patients. **a** Representative images of biopsies containing the expression of β-catenin and SIX1 in gastric cancer patient tissues with low and high expression of sf-RON and RON (× 400). **b**, **c** Correlation of sf-RON and RON expression with β-catenin and SIX1. **d–g** Kaplan-Meier OS survival curves (log-rank tests) and PFS survival curves (log-rank tests) of patients with varying sf-RON, RON, β-catenin, and SIX1 expression. **h** Schematic model showing the role of sf-RON/β-catenin/SIX1 signaling axis in the regulation of cell proliferation and glucose metabolism in the GC cells

To ascertain the prognostication value of sf-RON, β-catenin, and SIX1 in GC, we assessed their expression in GC tissues. Although the significance is low, we found the trend that the sf-RON and RON expression levels were positively correlated with those of β-catenin and SIX1 in GC (Fig. 6a–c). We also found that the patients with low sf-RON or RON expression levels had significantly better survival times compared to patients with high sf-RON/RON expression levels (Fig. 6d, e). Meanwhile, patients with low SIX1 and β-catenin expression levels had longer survival times than those with high expression of SIX1 and β-catenin (Fig. 6f, g). The correlation of sf-RON, β-catenin, and SIX1 with clinicopathological characteristics in GC patients was shown in Supplementary Table 4. Finally, we identify a sf-RON/β-catenin/SIX1 signaling axis in gastric cancer (Fig. 6h).

## Discussion

Here, we identified the regulatory role of sf-RON in the cell proliferation and glucose metabolism of GC. Mechanically, we demonstrated that sf-RON activated β-catenin/SIX1 signaling pathway to enhance glucose metabolism of GC cells, which caused GC cell proliferation. This is a noteworthy finding, for sf-RON may be recognized as a potential therapeutic target of GC and it offers a promising alternative route for cancer treatment.

We had reported the contribution of sf-RON in MET-positive GC, and it was a novel intrinsic resistant molecule of anti-MET therapy (Wu et al. 2015). However, the role of sf-RON in the proliferation and glucose metabolism of GC has not been discussed. In our study, we explored the contribution of sf-RON and RON to cancer cell metabolism in-depth. Our results demonstrated that sf-RON and RON regulated aerobic glycolysis, which is fundamental for facilitating cancer cell proliferation. RON is usually expressed together with one of its alternative transcription form sf-RON. Although the sf-RON protein is translated in-frame, it exerts a constitutively active receptor with a ligand-independent activity because of lacking the N-terminus and the ligand-binding domain of RON (Moxley et al. 2016). Given that sf-RON is an activated truncated form that shows constitutive kinase activity, so the deregulation of sf-RON may have stronger biological than the full-length RON in gastric cancer.

Aerobic glycolysis could provide cancerous cells with materials for macromolecule synthesis, and help to provide energy that favors uncontrolled proliferation. Furthermore, glycolytic genes in the cascade can play direct regulative roles in cancer cell behaviors. Our study demonstrated that SIX1, the well-known glycolytic and cell growth promoter, was a direct target of β-catenin to promote glucose metabolism in GC, and sf-RON can promote cell proliferation by increasing glucose metabolism via β-catenin/SIX1 signaling pathway. All these observations emphasized the crucial function of sf-RON in regulating GC malignant behavior. Besides, a previous report indicated that the SIX1 can be transcriptional regulated by a transducer of WNT/β-catenin signaling, TCF7L2 in acute myelocytic leukemia (Zhang et al. 2019), and WNT/β-catenin signaling promotes skeletal muscle development by increasing SIX1 levels (Petropoulos, Skerjanc 2002). However, SIX1 can also act as a DNA-specific transcription factor and recruit EYA1 to activate target gene expression, or interact with corepressor DACH1 to suppress transcription of target genes (Blevins et al. 2015). SIX1 was also reported to facilitate Warburg effect by upregulating many glycolytic genes (Li et al. 2018). What is more, SIX1 can also upregulate the well-known oncogene c-Myc and contribute to tumor growth in human cancer cells (Yu et al. 2006). All these emphasized the complicity and networked molecular mechanism of human bodies. Consistent with our results, SIX1 can inversely exert its oncogenic effect by activating Wnt/β-catenin signal (Song et al. 2019), which means that there may be a crosstalk between SIX1 and the Wnt/β-catenin pathway in the development of cancer.

Refer to β-catenin, it is a well-described protein which governs cell survival and growth in lots of diseases, such as gastrointestinal disorders (Michael et al. 2019; Chavesperez et al. 2019) and hepatocellular carcinoma (Metidji et al. 2018) as well as retinal diseases (Yao et al. 2018). Nevertheless, β-catenin is a multifunctional protein that can act both as an adaptor protein and a transcriptional co-regulator for intracellular adhesion, and causative to multiple growth-related pathologies (Clevers and Nusse 2012). For example, it can contribute to tumorigenesis by controlling stemness and suppresses the T cell responses (Shang et al. 2017). Thus, the RON/β-catenin might mediate proliferation and tumor growth independent of the glucose metabolism reprogramming. Though β-catenin has a close connection with cell proliferation, further data to elaborate that downregulation of metabolic genes by β-catenin may not simply result in a consequence of reduced cell viability would be important.

Moreover, as can be seen in lots of researches, chemotherapy is an effective treatment for GC (Shi and Gao 2016). In the previous study, it has been revealed that enhanced glucose metabolism remodeling is one of the fundamental characteristics of human tumor entities (Han et al. 2015), and is remarkably pivotal for the development of tumor therapy strategies and drug resistance (Song et al. 2016). Chemoresistant cancer cells are characterized by upregulated aerobic glycolysis (Firuzi et al. 2019), defective mitochondrial ATP production (Hawkes 2019), and increased absolute levels of intracellular ATP. Therefore, searching for ligands and signaling pathways that are linked to the chemoresistance faces challenges and it is worthwhile to devote much effort to this area. Our results verified the overexpression of sf-RON in GC tissues, and sf-RON could be an interesting candidate in cancer treatment. From the literature review, activation of the sf-RON signaling is one of the mechanisms underlying MET inhibitor unresponsiveness (Sehrawat and Singh 2016). Excessive activation of the MET axis was highly associated with chemoresistance in various human malignancies, including GC (Lin et al. 2019). Thus, a combination therapeutic strategy targeting both MET and sf-RON signaling may help to improve the efficacy of MET-targeted medicine (Maroun and Rowlands 2014). However, the role and underlying mechanism of sf-RON in the glucose metabolism remodeling remain need to be further explored. Our data elucidated that sf-RON considerably increased the glucose metabolism level of GC. We assumed that sf-RON may be increased in the MET-related drug resistance and promote regulation of glucose metabolism via the β-catenin/SIX1 signaling pathway.

Collectively, we substantiated the role of sf-RON in glucose metabolism-related cell growth, and we further illuminated the mechanism underlying β-catenin/SIX1 axis in GC cell proliferation. Therefore, sf-RON may warrant consideration as a valuable prognostic predictor for GC, and the RON/β-catenin/SIX1 axis could be a therapeutic target in prospective clinical studies.

## Electronic supplementary material

ESM 1 (DOCX 13 kb)

Supplementary Table 1 (DOC 37 kb)

Supplementary Table 2 (DOCX 17 kb)

Supplementary Table 3 (XLSX 10 kb)

Supplementary Table 4 (DOCX 18 kb)

Supplementary Figure 1 (PNG 623 kb)

High Resolution (TIF 1297 kb)

Supplementary Figure 2 (PNG 587 kb)

High Resolution (TIF 558 kb)

Supplementary Figure 3 (PNG 478 kb)

High Resolution (TIF 1214 kb)

## Data Availability

All data generated or analyzed during this study are included either in this article or in the supplementary materials and methods, tables, figures, and figure legends files.

## Abbreviations

AKT1: protein kinase B
ChIP: chromatin immunoprecipitation
ECAR: extracellular acidification rate
DFS: disease-free survival
FFPE: formalin-fixed and paraffin-embedded
FUSCC: Fudan University Shanghai Cancer Center
GC: gastric cancer
GLUT1: glucose
GSEA: gene set enrichment analysis
GSK3β: glycogen synthase kinase-3β
HEK: human embryonic kidney
HK2: hexokinase 2
TMA: tissue microarray analysis
IRS: immunoreactive score
LDHA: lactate dehydrogenase A
OCR: oxygen consumption rate
OS: overall survival
RTK: receptor tyrosine kinase
sf-RON: short-form Recepteur d’origine nantais
RON: Recepteur d’origine nantais
SIX1: Sine oculis homeobox homolog 1
SUVs: standardized uptake values
