# Supplementary material for: Short-form RON (sf-RON) enhances glucose metabolism to promote cell proliferation via activating β-catenin/SIX1 signaling pathway in gastric cancer
Source: Cell Biol Toxicol. 2020 May 12;37(1):35–49. doi: 10.1007/s10565-020-09525-5 (PMC7851020; doi:10.1007/s10565-020-09525-5)
Supplement: Supplementary file 1 — (DOCX 13 kb) [file 10565_2020_9525_MOESM1_ESM.docx]

**Supplementary Figure Legends**

**Supplementary Figure 1. sf-RON enhanced the proliferation of MKN-45 and GTL-16 cells by targeting β-catenin**

**A-B,** Overexpression of sf-RON promoted cell growth and increased the number and size of the colonies in sf-RON overexpressing cells compared with control cells.

**Supplementary Figure 2. Silencing of sf-RON and RON caused the decrease of the glycolysis in GC cells.**

**A,** Silencing of sf-RON and RON was detected by Western blotting assay. **B,** Silencing of sf-RON and RON inhibited cell growth in MKN-45 and GTL-16 cell lines compared with control cells using CCK-8 kit. **C,** Silencing of sf-RON and RON inhibited glucose uptake, lactate uptake and ATP uptake dramatically in MKN-45 and GTL-16 cell lines when compared with controls. **D-E,** Silencing of sf-RON and RON inhibited extracellular acidification rate (ECAR) and oxygen consumption rate (OCR) dramatically in gastric cells when compared with controls. Error bars = 95% CIs. The experiments were repeated three times, and a representative experiment is shown. F, Silencing of sf-RON and RON decreased the expression levels of GLUT1, LDHA and HK2 by Western blotting assay

**Supplementary Figure 3. Introduction of SIX1 cDNA rescued the effects on glucose metabolism affected by the silencing of β-catenin in sf-RON-overexpressing gastric cells**

**A-B,** Overexpression of SIX1 rescued the effects on the expression of GLUT1 and LDHA affected by the silencing of β-catenin in sf-RON-overexpressing gastric cells by Western blotting assay. **C-F,** Introduction of SIX1 cDNA into β-catenin-silenced cells restored the glucose consumption, glucose uptake, lactate uptake and ATP uptake levels of these cells, compared with the control cells. **G,** Introduction of SIX1 cDNA into β-catenin-silenced cells restored on cell proliferation detected by CCK-8 kit assay.
