# Supplementary material for: Short-form RON (sf-RON) enhances glucose metabolism to promote cell proliferation via activating β-catenin/SIX1 signaling pathway in gastric cancer
Source: Cell Biol Toxicol. 2020 May 12;37(1):35–49. doi: 10.1007/s10565-020-09525-5 (PMC7851020; doi:10.1007/s10565-020-09525-5)
Supplement: Supplementary file 2 — (DOC 37 kb) [file 10565_2020_9525_MOESM2_ESM.doc]

**Supplementary Table 1.** **Univariate and multivariate analyses of clinicopathological factors for overall survival in gastric cancer patients**

| Variable | Univariate analysis | | | Multivariate analysis | |  |
| --- | --- | --- | --- | --- | --- | --- |
|  | HR (95 % CI) | p^a^ | HR (95 % CI) | | p^a^ | |
| Age | 1.007 (0.983-1. 033) | 0.563 |  | |  | |
| Gender | 1.374 (0.782-2.414) | 0.269 |  | |  | |
| Lauren classification | 1.332 (1.875-2.027) | 0.181 |  | |  | |
| Histologic grade | 0.777 (0.434-1.391) | 0.396 |  | |  | |
| Vascular invasion | 1.747 (1.004-3.042) | **0.049** |  | |  | |
| Nervous invasion | 1.620 (0.937-2.798) | 0.084 |  | |  | |
| TNM stage | 2.487 (1.582-3.909) | **< 0.001** |  | |  | |
| sf-RON protein | 6.318 (1.353-29.493) | **0.019** |  | |  | |
| RON protein | 2.463 (1.475-4.112) | **0.001** | 5.785 (1.345-24.886) | | **0.018** | |
| CTNNB1 protein | 1.691 (1.007-2.837) | **0.047** |  | |  | |
| SIX1 protein | 2.462 (1.264-4.796) | **0.008** |  | |  | |

HR: Hazard ratio; CI: confidence interval; ^a^ All statistical tests were 2-sided. Significance level: p < 0.05.
