# Supplementary material for: Short-form RON (sf-RON) enhances glucose metabolism to promote cell proliferation via activating β-catenin/SIX1 signaling pathway in gastric cancer
Source: Cell Biol Toxicol. 2020 May 12;37(1):35–49. doi: 10.1007/s10565-020-09525-5 (PMC7851020; doi:10.1007/s10565-020-09525-5)
Supplement: Supplementary file 3 — (DOCX 17 kb) [file 10565_2020_9525_MOESM3_ESM.docx]

**Supplementary Table 2 . The primer sequences of qRT-PCR assay**

| Gene | Number | Primer |
| --- | --- | --- |
| β-catenin | F | GTGGGGACAAGTCGTCTGG |
|  | R | AGTGCTGGCATCGGTTTCG |
| SIX1 | F | TGGCATTTGCTGAACGCATTT |
|  | R | TGCAGCCAGGTCTAATTGTTTT |
| GLUT1 | F | GAGGTAGTCAGACGGAAGCTA |
|  | R | GAGGGGAACATGCAGTCATTT |
| LDHA | F | ATGGCAACTCTAAAGGATCAGC |
|  | R | CCAACCCCAACAACTGTAATCT |
| ALDOA | F | GTTATCAAATCCAAGGGCGGTG |
|  | R | AGTCAGCTCCGTCCTTCTTGTAC |
| ENO1 | F | GCTCCGGGACAATGATAAGACTCG |
|  | R | CTGTTCCATCCATCTCGATCATC |
| PKM2 | F | CAAAGGACCTCAGCAGCCATGTC |
|  | R | GGGAAGCTGGGCCAATGGTACAGA |
| PGK1 | F | CAAGGTTAAAGCCGAGCCAGCC |
|  | R | GCCTTCTGTGGCAGATTGACTC |
| HK2 | F | GATTGTCCGTAACATTCTCATCG |
|  | R | TGTCTTGAGCCGCTCTGAGAT |
| GAPDH | F | GGCCTCCAAGGAGTAAGACC |
|  | R | CAAGGGGTCTACATGGCAAC |
