# Supplementary material for: Short-form RON (sf-RON) enhances glucose metabolism to promote cell proliferation via activating β-catenin/SIX1 signaling pathway in gastric cancer
Source: Cell Biol Toxicol. 2020 May 12;37(1):35–49. doi: 10.1007/s10565-020-09525-5 (PMC7851020; doi:10.1007/s10565-020-09525-5)
Supplement: Supplementary file 5 — (DOCX 18 kb) [file 10565_2020_9525_MOESM5_ESM.docx]

**Supplementary Table 4. The relationship between clinicopathological characteristics and RON, sfRON, CTNNB1 and SIX1 expression in gastric cancer patients**

| Variables | sfRON-low（%） | sfRON-high（%） | P ^a^ | RON-low（%） | RON-high（%） | P ^a^ | β-catenin-low （%） | β-catenin-high （%） | P ^a^ | SIX1-low （%） | SIX1-high（%） | P ^a^ |
| --- | --- | --- | --- | --- | --- | --- | --- | --- | --- | --- | --- | --- |
| Age |  |  | 0.695 |  |  | 0.118 |  |  | 0.745 |  |  | 0.860 |
| ≤60 | 8 (61.5) | 6 (46.2) |  | 57 (53.8%) | 34 (68.0%) |  | 47 (56.6) | 44 (60.3) |  | 29 (60.4) | 62 (57.4) |  |
| ＞60 | 5 (38.5) | 7 (53.8) |  | 49 (46.2) | 16 (32.0) |  | 36 (43.4) | 29 (39.7) |  | 19 (39.6) | 46 (42.6) |  |
| Gender |  |  | 0.678 |  |  | 0.318 |  |  | 0.577 |  |  | 0.901 |
| male | 10 (76.9) | 10 (76.9) |  | 83 (78.3) | 35 (70.0) |  | 61 (73.5) | 57 (78.1) |  | 36 (75.0) | 82 (75.9) |  |
| female | 3 (23.1) | 3 (23.1) |  | 23 (21.7) | 15 (30.0) |  | 22 (26.5) | 16 (21.9) |  | 12 (25.0) | 26 (24.1) |  |
| Lauren classification |  |  | 0.500 |  |  | 0.071 |  |  | 0.345 |  |  | 0.189 |
| Intestinal | 10 (76.9) | 11 (84.6) |  | 73 (68.9) | 27 (54.0) |  | 51 (61.4) | 49 (67.1) |  | 30 (62.5) | 70 (64.8) |  |
| Diffuse | 3 (23.1) | 2 (15.4) |  | 31 (29.2) | 19 (38.0) |  | 30 (36.1) | 20 (27.4) |  | 18 (37.5) | 32 (29.6) |  |
|  |  |  |  | 2 (1.9) | 4 (8.0) |  | 2 (2.4) | 4 (5.5) |  | 0 (0.0) | 6 (5.6) |  |
| Histologic grade |  |  | **0.015** |  |  | 0.281 |  |  | 0.817 |  |  | 0.450 |
| Good |  |  |  | 1 (0.9) | 0 (0.0) |  | 0 (0.0) | 1 (1.4) |  | 0 (0.0) | 1 (0.9) |  |
| moderate | 0 (0.0) | 7 (53.8) |  | 48 (45.3) | 12 (24.0) |  | 11 (13.3) | 10 (13.7) |  | 7 (14.6) | 14 (13.0) |  |
| Poor | 11(83.8) | 6 (46.2) |  | 52 (49.1) | 38 (76.0) |  | 70 (84.3) | 59 (80.9) |  | 38 (79.2) | 91 (84.3) |  |
| Undifferentiation | 2 (15.4) | 0 (0.0) |  | 5 (4.7) | 0 (0.0) |  | 2 (2.4) | 3 (4.1) |  | 3 (6.3) | 2 (1.9) |  |
| Vascular invasion |  |  | 0.691 |  |  | **0.023** |  |  | 0.519 |  |  | 0.914 |
| Absent | 6 (46.2) | 5 (38.5) |  | 46 (43.4) | 18 (36.0) |  | 32 (38.6) | 32 (43.8) |  | 20 (41.7) | 44 (40.7) |  |
| Present | 7 (53.8) | 8 (61.5) |  | 60 (56.6) | 32 (64.0) |  | 51 (61.4) | 41 (56.2) |  | 28 (58.3) | 64 (59.3) |  |
| Nervous invasion |  |  | 0.688 |  |  | 0.051 |  |  | 0.872 |  |  | 0.729 |
| Absent | 7 (53.8) | 9 (69.2) |  | 48 (45.3) | 17 (34.0) |  | 34 (41.0) | 31 (42.5) |  | 21 (43.8) | 44 (40.7) |  |
| Present | 6 (46.2) | 4 (30.8) |  | 58 (54.7) | 33 (66.0) |  | 49 (59.0) | 42 (57.5) |  | 27 (56.3) | 64 (59.3) |  |
| TNM stage |  |  | 0.589 |  |  | 0.756 |  |  | 0.181 |  |  | 0.683 |
| I |  |  |  | 6 (5.7) | 2 (4.0) |  | 5 (6.0) | 3 (4.1) |  | 4 (8.3) | 4 (3.7) |  |
| II | 4 (30.8) | 4 (30.8) |  | 25 (23.6) | 14 (28.0) |  | 15 (18.1) | 24 (32.9) |  | 12 (25.0) | 27 (25.0) |  |
| III | 8 (69.2) | 8 (61.5) |  | 64 (60.4) | 31 (62.0) |  | 54 (65.1) | 41 (56.2) |  | 28 (58.3) | 67 (62.0) |  |
| IV | 0 (0) | 1 (7.7) |  | 11 (10.4) | 3 (6.0) |  | 9 (10.8) | 5 (6.8) |  | 4 (8.3) | 10 (9.3) |  |
| Recurrence |  |  | **0.047** |  |  | **0.003** |  |  | 0.147 |  |  | **0.009** |
| Absent | 9 (69.2) | 3 (23.1) |  | 68 (64.2) | 19 (38.0) |  | 51 (61.4) | 36 (49.3) |  | 34 (70.8) | 53 (49.1) |  |
| Present | 4 (30.8) | 10 (76.9) |  | 38 (35.8) | 31 (62.0) |  | 32 (38.6) | 37 (50.7) |  | 14 (29.2) | 55 (50.9) |  |

(Two-sided χ2 test or Fisher’s exact test for distributions between negative and positive expression of sf-RON, RON, β-catenin and SIX1.
