# Supplementary figures and images for: Short-form RON (sf-RON) enhances glucose metabolism to promote cell proliferation via activating β-catenin/SIX1 signaling pathway in gastric cancer
Source: Cell Biol Toxicol. 2020 May 12;37(1):35–49. doi: 10.1007/s10565-020-09525-5 (PMC7851020; doi:10.1007/s10565-020-09525-5)

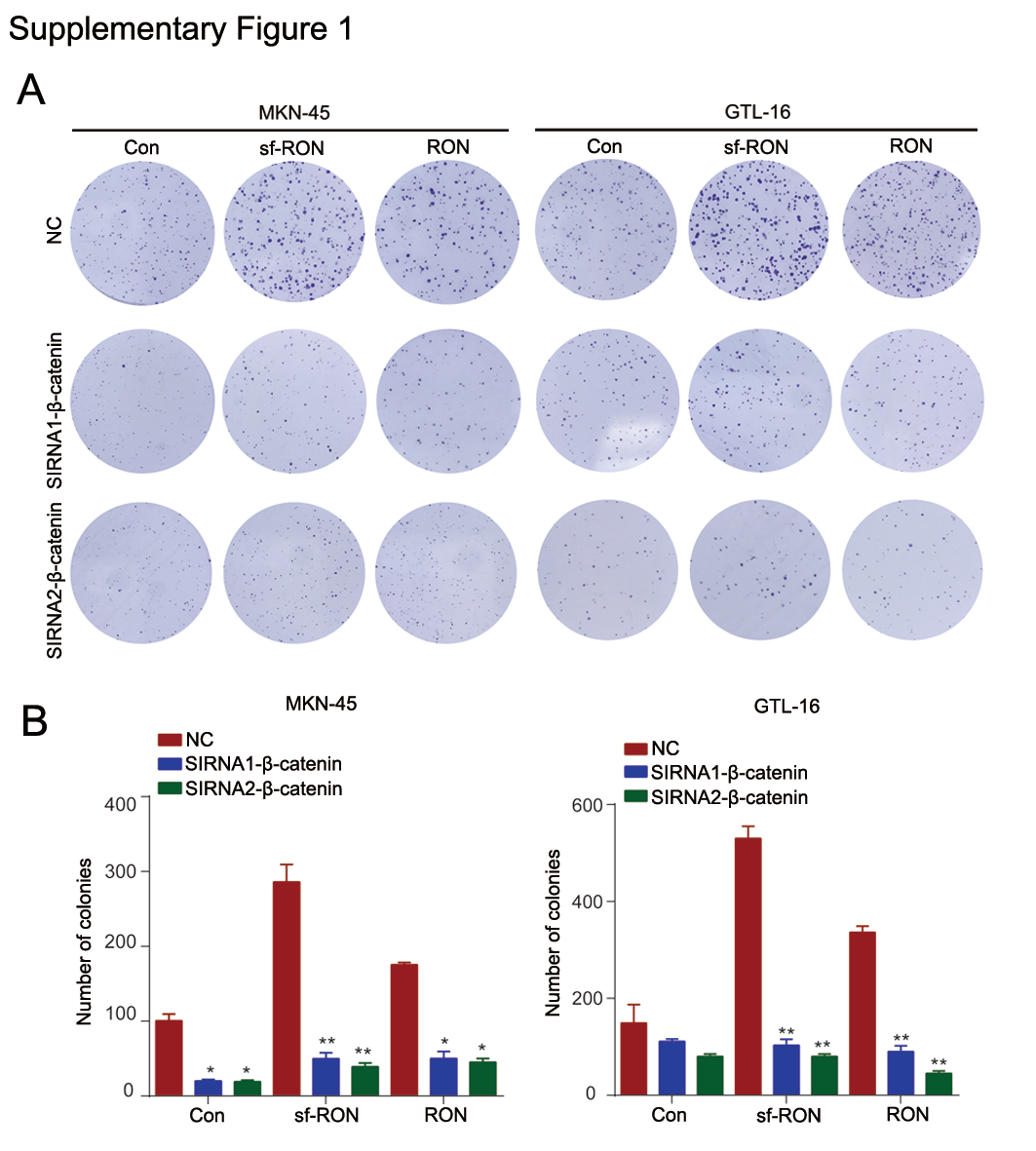

Supplement: Supplementary file 6 — (PNG 623 kb) [file 10565_2020_9525_Fig7_ESM.png]

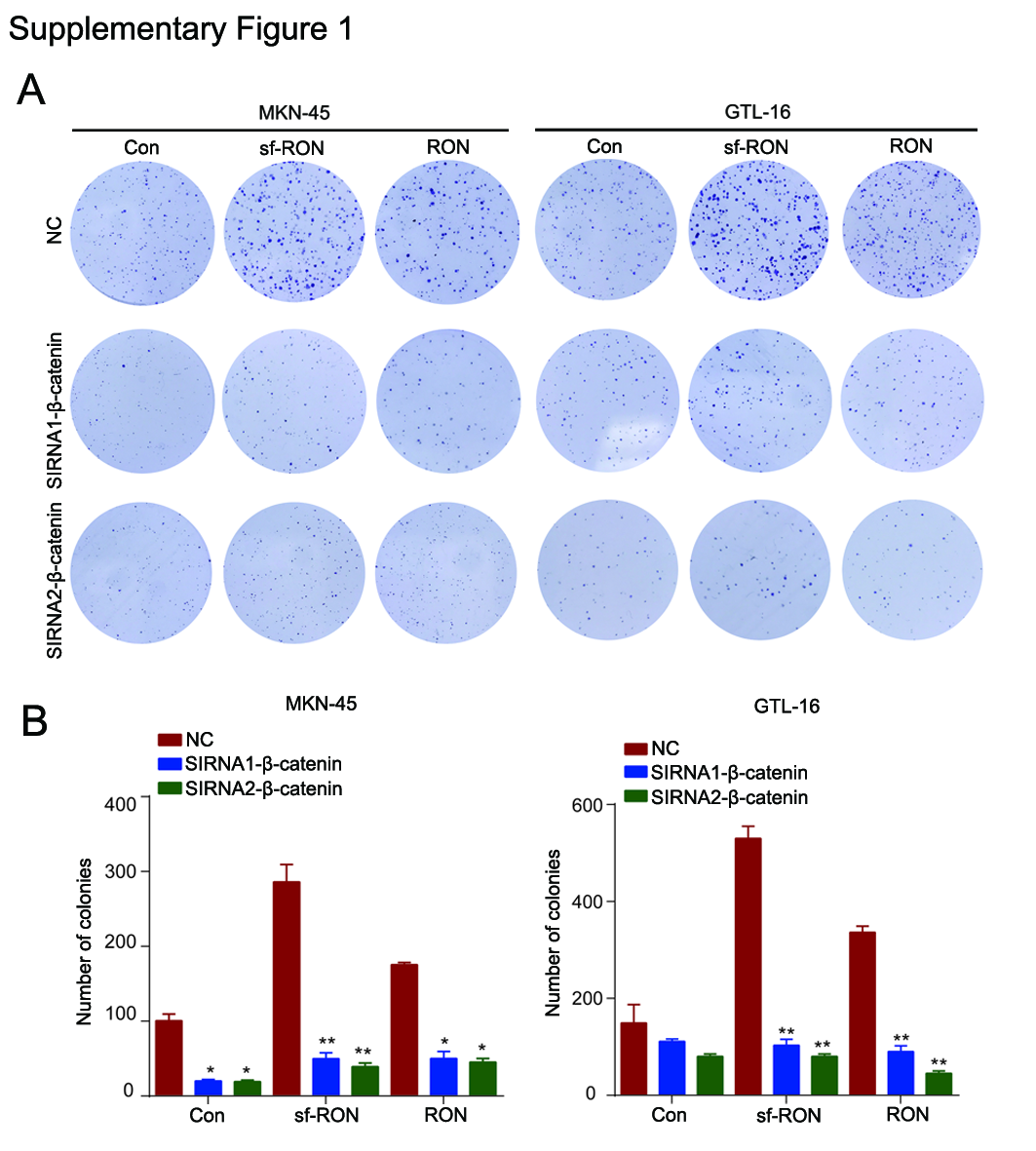

Supplement: Supplementary file 7 — High Resolution (TIF 1297 kb) [file 10565_2020_9525_MOESM6_ESM.tif]

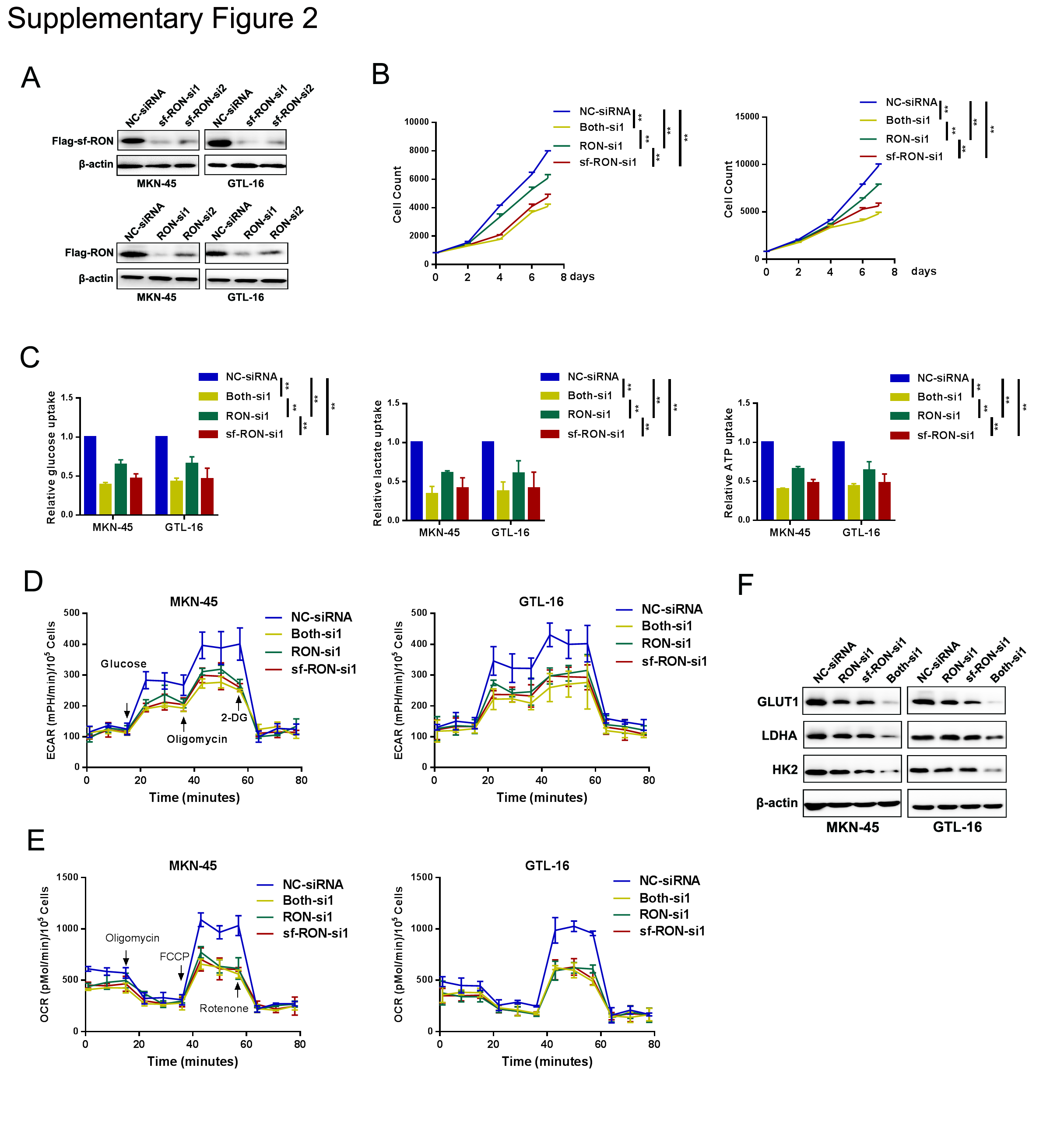

Supplement: Supplementary file 8 — (PNG 587 kb) [file 10565_2020_9525_Fig8_ESM.png]

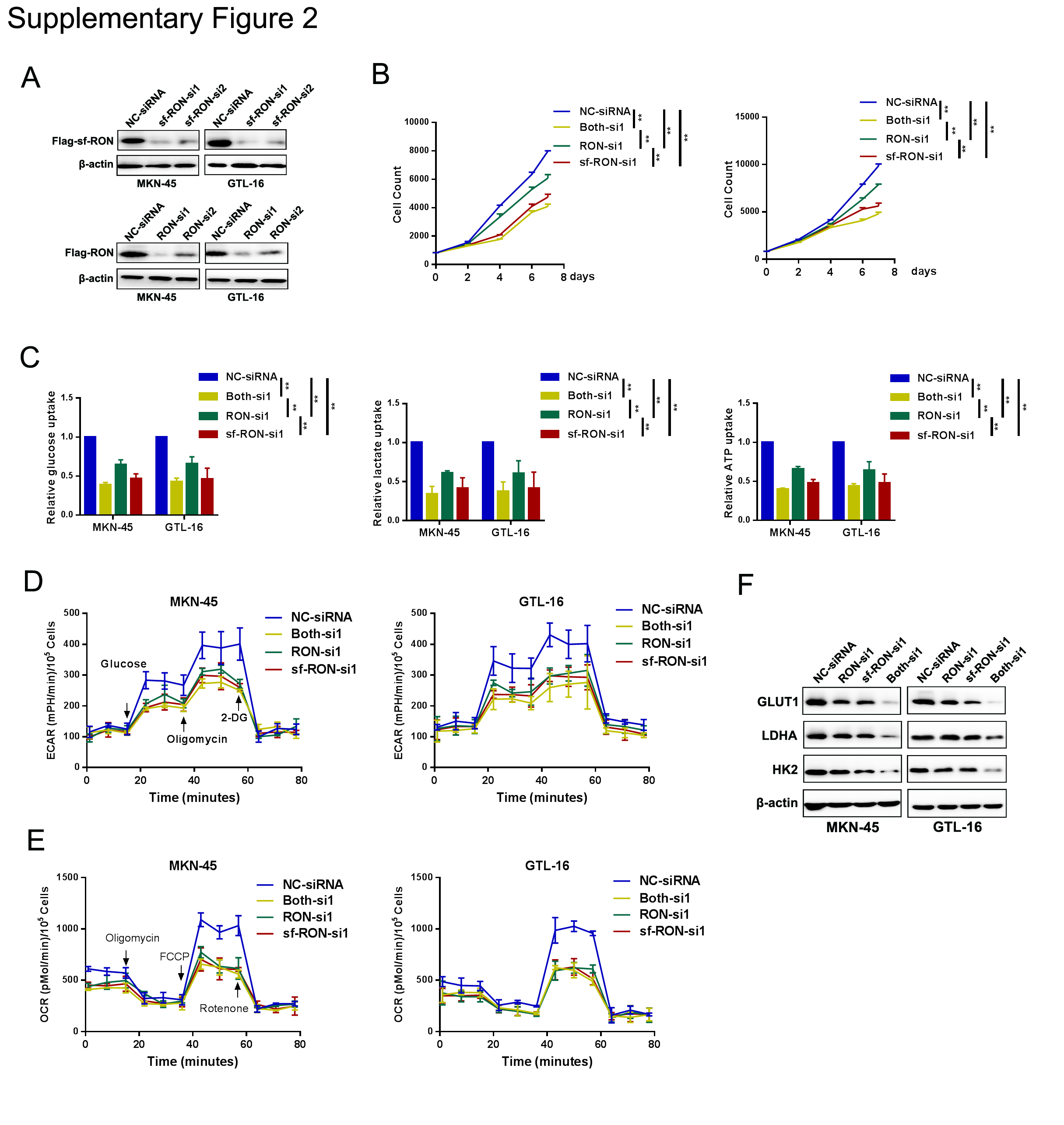

Supplement: Supplementary file 9 — High Resolution (TIF 558 kb) [file 10565_2020_9525_MOESM7_ESM.tif]

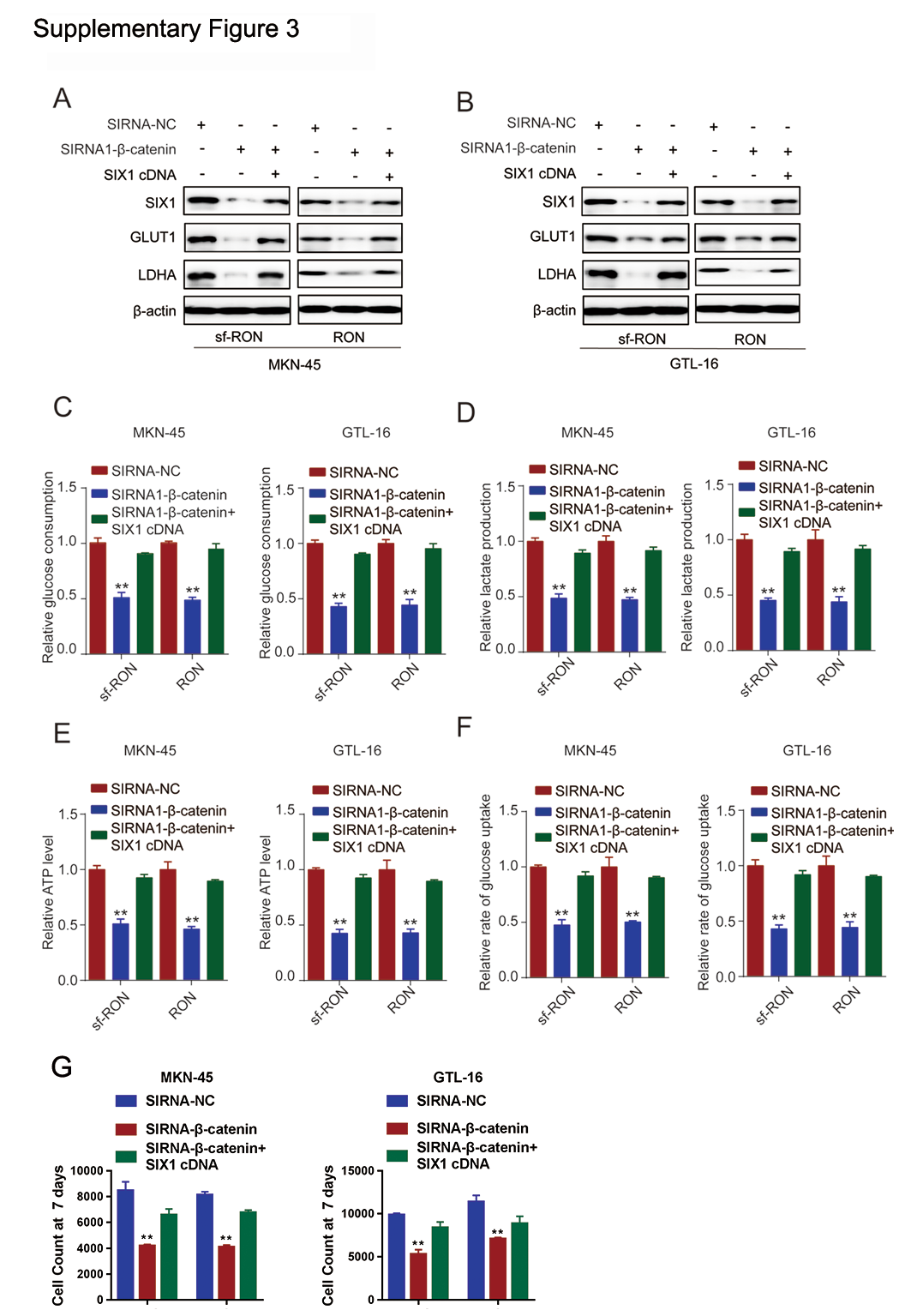

Supplement: Supplementary file 10 — (PNG 478 kb) [file 10565_2020_9525_Fig9_ESM.png]

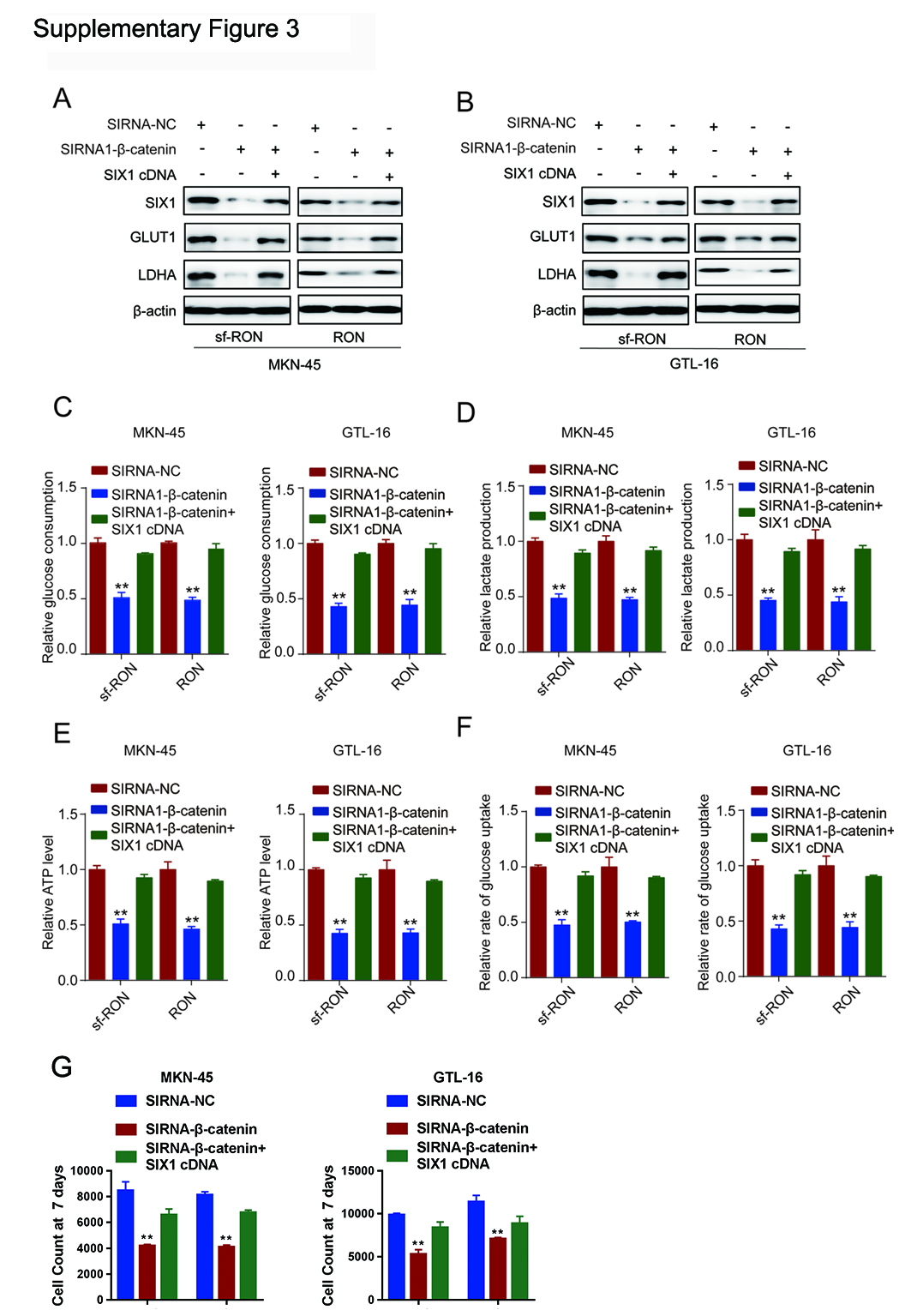

Supplement: Supplementary file 11 — High Resolution (TIF 1214 kb) [file 10565_2020_9525_MOESM8_ESM.tif]
